# Prediction of breast cancer risk based on common genetic variants in women of East Asian ancestry

**DOI:** 10.1186/s13058-016-0786-1

**Published:** 2016-12-08

**Authors:** Wanqing Wen, Xiao-ou Shu, Xingyi Guo, Qiuyin Cai, Jirong Long, Manjeet K. Bolla, Kyriaki Michailidou, Joe Dennis, Qin Wang, Yu-Tang Gao, Ying Zheng, Alison M. Dunning, Montserrat García-Closas, Paul Brennan, Shou-Tung Chen, Ji-Yeob Choi, Mikael Hartman, Hidemi Ito, Artitaya Lophatananon, Keitaro Matsuo, Hui Miao, Kenneth Muir, Suleeporn Sangrajrang, Chen-Yang Shen, Soo H. Teo, Chiu-chen Tseng, Anna H. Wu, Cheng Har Yip, Jacques Simard, Paul D. P. Pharoah, Per Hall, Daehee Kang, Yongbing Xiang, Douglas F. Easton, Wei Zheng

**Affiliations:** 1 Division of Epidemiology, Department of Medicine, Vanderbilt University School of Medicine, Nashville, TN USA; 2 Centre for Cancer Genetic Epidemiology, Department of Public Health and Primary Care, University of Cambridge, Cambridge, UK; 3 Department of Epidemiology, Shanghai Cancer Institute, Shanghai, China; 4 Shanghai Municipal Center for Disease Control and Prevention, Shanghai, China; 5 Centre for Cancer Genetic Epidemiology, Department of Oncology, University of Cambridge, Cambridge, UK; 6 Division of Genetics and Epidemiology, The Institute of Cancer Research, London, UK; 7 Division of Cancer Epidemiology and Genetics, National Cancer Institute, Rockville, MD USA; 8 International Agency for Research on Cancer, Lyon, France; 9 Department of Surgery, Changhua Christian Hospital, Changhua, Taiwan; 10 Department of Biomedical Sciences, Seoul National University College of Medicine, Seoul, Korea; 11 Cancer Research Institute, Seoul National University, Seoul, Korea; 12 Saw Swee Hock School of Public Health, National University of Singapore, Singapore, Singapore; 13 Department of Surgery, National University Health System, Singapore, Singapore; 14 Division of Epidemiology and Prevention, Aichi Cancer Center Research Institute, Nagoya, Japan; 15 Division of Health Sciences, Warwick Medical School, Warwick University, Coventry, UK; 16 Division of Molecular Medicine, Aichi Cancer Center Research Institute, Nagoya, Japan; 17 Institute of Population Health, University of Manchester, Manchester, UK; 18 National Cancer Institute, Bangkok, Thailand; 19 School of Public Health, China Medical University, Taichung, Taiwan; 20 Taiwan Biobank, Institute of Biomedical Sciences, Academia Sinica, Taipei, Taiwan; 21 Cancer Research Initiatives Foundation, Subang Jaya, Selangor, Malaysia; 22 Breast Cancer Research Unit, Cancer Research Institute, University Malaya Medical Centre, Kuala Lumpur, Malaysia; 23 Department of Preventive Medicine, Keck School of Medicine, University of Southern California, Los Angeles, CA USA; 24 Genomics Center, Centre Hospitalier Universitaire de Québec Research Center, Laval University, Québec City, Canada; 25 Department of Medical Epidemiology and Biostatistics, Karolinska Institutet, Stockholm, Sweden; 26 Department of Preventive Medicine, Seoul National University College of Medicine, Seoul, Korea; 27 Vanderbilt Epidemiology Center and Vanderbilt-Ingram Cancer Center, Vanderbilt University School of Medicine, 2525 West End Avenue, 8th Floor, Nashville, TN 37203-1738 USA

**Keywords:** Breast cancer risk, Prediction model, Methodology for SNP data analysis, Statistical methods in genetics

## Abstract

**Background:**

Approximately 100 common breast cancer susceptibility alleles have been identified in genome-wide association studies (GWAS). The utility of these variants in breast cancer risk prediction models has not been evaluated adequately in women of Asian ancestry.

**Methods:**

We evaluated 88 breast cancer risk variants that were identified previously by GWAS in 11,760 cases and 11,612 controls of Asian ancestry. SNPs confirmed to be associated with breast cancer risk in Asian women were used to construct a polygenic risk score (PRS). The relative and absolute risks of breast cancer by the PRS percentiles were estimated based on the PRS distribution, and were used to stratify women into different levels of breast cancer risk.

**Results:**

We confirmed significant associations with breast cancer risk for SNPs in 44 of the 78 previously reported loci at *P* < 0.05. Compared with women in the middle quintile of the PRS, women in the top 1% group had a 2.70-fold elevated risk of breast cancer (95% CI: 2.15–3.40). The risk prediction model with the PRS had an area under the receiver operating characteristic curve of 0.606. The lifetime risk of breast cancer for Shanghai Chinese women in the lowest and highest 1% of the PRS was 1.35% and 10.06%, respectively.

**Conclusion:**

Approximately one-half of GWAS-identified breast cancer risk variants can be directly replicated in East Asian women. Collectively, common genetic variants are important predictors for breast cancer risk. Using common genetic variants for breast cancer could help identify women at high risk of breast cancer.

**Electronic supplementary material:**

The online version of this article (doi:10.1186/s13058-016-0786-1) contains supplementary material, which is available to authorized users.

## Background

Genome-wide association studies (GWAS) to date have identified approximately 100 genetic loci associated with breast cancer risk [1–12]. Approximately 10 of these loci were initially identified in GWAS conducted in East Asian descendants [7–12]. Virtually all other loci were initially identified in studies conducted with European descendants. In a recent study, we confirmed a significant association in East Asian women for 31 of the 67 independent breast cancer susceptibility loci reported from previous GWAS conducted mostly in European descendants [13]. Previously we constructed an eight-SNP polygenic risk score (PRS) and found it to be the third strongest predictor for breast cancer risk, behind waist-to-hip ratio and previous benign breast disease. Adding the PRS to a predictive model including these two risk factors increases the area under the receiver operating characteristic curve (AUC) from 0.6178 to 0.6295 [7]. More recently, a relatively small study with 411 breast cancer cases and 1212 controls conducted in Singapore Chinese participants reported that a PRS constructed from 51 SNPs improved the classification of 6.2% of the women for their absolute risk of breast cancer in the next 5 years [14].

We have recently identified several new genetic variants associated with breast cancer risk among women of Asian ancestry [8–12]. As more breast cancer risk-related genetic variants are found, it is important to investigate the public health impact of those genetic variants to identify susceptible subgroups of individuals at elevated breast cancer risk to provide cost-efficient prevention strategies and to make appropriate healthcare decisions. In this study, we investigate the value of genetic information in predicting breast cancer risk in women of East Asian ancestry.

## Methods

### Study populations

This study gathered data from 11 participating case–control studies from three sources: 12,893 women (6269 cases and 6624 controls) of East Asian origin participating in nine studies in the Breast Cancer Association Consortium (BCAC) that were conducted in China, Japan, South Korea, Thailand, and Malaysia; 5152 Chinese women (2867 cases and 2285 controls) from the Shanghai Genome-Wide Association Studies (SGWAS) who were participants in the Shanghai Breast Cancer Study (SBCS), the Shanghai Breast Cancer Survival Study (SBCSS), and the Shanghai Women’s Health Study (SWHS) (the SBCS is a population-based case–control study, and the SBCSS and SWHS are ongoing population-based, prospective cohort studies—all participants in these studies were recruited in Shanghai during the same time period from 1996 to 2005 using similar study protocols); and 5522 Chinese women (2769 cases and 2753 controls) who were participants in Stage 2 of the Shanghai breast cancer Genome-Wide Association Studies (SGWAS-stage2) [11]. In total, 23,567 women of East Asian ancestry (11,905 cases and 11,662 controls) were included in the current analysis (Additional file 1: Table S1). All participating studies obtained written, informed consent from all subjects and approval from their respective Institutional Review Boards. No participant received a stipend.

### Genotyping methods

Samples from the nine studies in the BCAC were genotyped using a custom Illumina iSelect array (iCOGS) comprising 211,155 SNPs, as part of a large collaboration for replication and fine-mapping of promising associations selected from GWAS of multiple cancers. Detailed information about the quality control (QC) has been described previously [5, 13]. Briefly, SNPs which had a call rate < 95%, deviated from Hardy–Weinberg equilibrium in controls at *P* < 10^−7^, or had genotype discrepancies in >2% of duplicate samples were excluded across all Collaborative Oncological Gene–environment Study (COGS) consortia.

The SGWAS samples were genotyped using Affymetrix 6.0, comprising 906,602 SNPs, and Affymetrix 500 K array, comprising approximately 500,000 SNPs [7]. Genetically identical and unexpected duplicate samples were excluded, as were close relatives with a pairwise proportion of identify-by-descent estimate > 0.25. All samples with a call rate < 95% were excluded. SNPs were excluded if the minor allele frequency was <1% or the genotyping concordance rate was <95% in the QC sample.

The SGWAS-stage2 samples were genotyped using an exome chip comprising approximately 50,000 SNPs with minor allele frequency over 1%, which included most of the GWAS-identified breast cancer variants [11].

Most SNPs included in this analysis were genotyped directly, and some SNPs were imputed using IMPUTE and the 1000 Genomes data as a reference panel.

### Statistical methods

A total of 88 SNPs at 78 breast cancer loci identified to date were included in this analysis. First, we evaluated associations between each SNP and breast cancer risk using logistic regression, assuming a log-additive genetic model with adjustment for age, population structure (principal components), and study sites, when applicable. We analyzed the association between each SNP and breast cancer risk separately for each data source. The final associations, combining the three sources, were derived using fixed-effect meta-analysis with inverse-variance weights. Any SNP with an association *P* < 0.05 (one-sided) was considered statistically significant. Tests for pairwise SNP by SNP interactions were also evaluated using logistic regression under the log-additive genetic model with the same adjustments already stated.

Second, to investigate the association between breast cancer risk and the combined effects of all significant SNPs, a PRS was derived for each study participant using the formula:

1 $$ PRS={\sum}_{i=0}^n{\beta}_iSN{P}_i $$

where *β*_*i*_ is the per-allele log odds ratio (OR) for breast cancer associated with the risk allele for *SNP*_*i*_, which is the number of risk alleles (0, 1, or 2) for the SNP, and *n* is the total number of significant SNPs. Thus, the PRS summarizes the combined effect of SNPs having significant association with breast cancer risk.

Under the multiplicative polygenic model, and given a large number of unlinked loci, each conferring a small effect, the population distribution of the PRS is normal (*F* = *N*(*μ*, *σ* ^2^)), with mean value *μ* and variance *σ* ^2^ [15, 16]:

2 $$ \mu =2{\sum}_i{p}_i{\beta}_i $$

3 $$ {\sigma}^2={\sum}_i{\sigma}_i^2=2{\sum}_i{p}_i{q}_i{\beta}_i^2 $$

where *p*_*i*_ is the effect allele frequency of the SNP_*i*_, *q*_*i*_ = 1 − *p*_*i*_, and β_*i*_ is the log OR.

The distribution of the PRS in breast cancer cases is also normal (*G* = *N*(*μ*', *σ*'^2^)), with the parameters *μ*' = *μ* + *σ* ^2^ and *σ*'^2^ = *σ* ^2^ [15, 16].

Third, the discriminative accuracy of using the PRS to predict breast cancer risk was evaluated with the AUC, which was calculated theoretically [17, 18] given that the PRS distributions (*F, G*) are known:

4 $$ AUC={\displaystyle {\int}_0^1\left(1-G(r)\right)dF(r)} $$

Additionally, the AUC was also evaluated using logistic regression models and a nonparametric approach [19]. The AUC does not measure risk concentration, which was evaluated with the proportion of cases followed (PCF), as the proportion of cases that would be followed in a program that followed the proportion *q* of the population at highest risk. The proportion *q* is the complementary measure, the proportion needed to follow-up (PNF) [17, 18]. Given PNF and the PRS distributions (*F, G*):

5 $$ PCF(q)=\Phi \left(\frac{\left({\Phi}^{-1}(q)\sigma +\mu \right)-\mu \hbox{'}}{\sigma}\right) $$

Finally, we used an approach similar to that described previously for the Gail model [20] to estimate the absolute risk of breast cancer according to percentile of the PRS. Specifically, we predicted the probability of developing breast cancer between ages α and α + τ for a woman who is in PRS percentile *j* as:

6 $$ P\left(\alpha, \tau, O{R}_j(t)\right)={\displaystyle {\int}_{\alpha}^{\alpha +\tau }{h}_1(t)O{R}_j}(t) \exp \left[-{\displaystyle {\int}_{\alpha}^{\tau}\left({h}_1(u)O{R}_j(u)+{h}_2(u)\right)du}\right]dt $$

where subscript 1 refers to the incidence of breast cancer and subscript 2 refers to all other causes of death. In Eq. (6), *h*_1_(*t*) is the baseline hazard rate of developing breast cancer at age *t* in the reference group, *h*_1_(*t*) = *h* ^*^(*t*)(1 – PAR), where PAR is the population attributable risk (PAR) related to the PRS and the theoretical prediction of the OR_*j*_ for individuals in the PRS interval *j* between two percentiles (*u, v*) versus the 40th–60th percentiles:

7 $$ O{R}_j=\frac{\left(0.6-0.4\right)\left(\Phi \left({\Phi}^{-1}\left(1-u\right)+\sigma \right)-\Phi \left({\Phi}^{-1}\left(1-v\right)+\sigma \right)\right)}{\left(v-u\right)\left(\Phi \left({\Phi}^{-1}(0.6)+\sigma \right)-\Phi \left({\Phi}^{-1}(0.4)+\sigma \right)\right)} $$

8 $$ PAR=1-{\displaystyle \sum}\frac{\Phi \left({\Phi}^{-1}\left(1-u\right)+\sigma \right)-\Phi \left({\Phi}^{-1}\left(1-v\right)+\sigma \right)}{O{R}_j} $$

and *h* ^*^(*t*) is the age-specific breast cancer incidence rate in a composite population, in urban Shanghai during 2002 and 2003 [21] or in Korean women in the Korean risk assessment model for breast cancer risk prediction [22]; and *h*_*2*_(*t*) is the mortality rate at age *t* from all causes of death, except breast cancer, in the population, estimated using age-specific nonbreast cancer mortality in Shanghai in 2002 and 2003 [21] or in Korean women [22].

## Results

The association between the 88 selected SNPs at the 78 genetic loci and breast cancer risk in East Asian women are presented in Additional file 2: Table S2, Additional file 3: Table S3, and Additional file 4: Table S4. Of those 78 loci, we observed 44 independent genetic loci that were significantly associated with breast cancer risk at *P* < 0.05 (one-sided, Additional file 2: Table S2, Additional file 3: Table S3, and Additional file 4: Table S4). We did not observe significant heterogeneity (data not shown) of the association across participating studies. No significant association with breast cancer risk was observed for the other 34 loci.

The PRS was derived based on the effect (*β*) and the number of risk alleles of a SNP carried by a woman. Some loci had multiple SNPs. In three of these loci (near *C6orf97*, *ZNF365*, and *ANKLE1* genes), the most significant SNPs in Asian women (rs2046210, rs10822013, and rs2363956) were different from the most significant SNPs in European women (rs3757318, rs10995190, and rs8170). Only the SNP with the most significant association with breast cancer risk in each locus was selected for the PRS. The PRS for Asian women therefore included 44 SNPs.

Under the multiplicative polygenic model, we observed a standard deviation (SD) of 0.38 for the PRS distribution in East Asian women (Eq. (3)). The theoretically predicted ORs from Eq. (4) and the observed ORs from logistic regression models for different percentiles of the PRS were compared with women in the 40th–60th percentiles (Table 1). The predicted and the observed estimates for ORs were similar, which provides support for the multiplicative polygenic model. Compared with Asian women in the middle quintile, for Asian women in the highest 1% of the PRS the theoretically predicted OR was 2.77 and the observed OR was 2.70 (95% CI: 2.15–3.40); for Asian women in the lowest 1% of the PRS, the theoretically predicted OR was 0.37 and the observed OR was 0.39 (95% CI: 0.27–0.57). The OR for the increment per decile of PRS was 1.13.

**Table 1:** Theoretically predicted OR and observed OR (95% CI) by the PRS percentiles

PRS (%)	Predicted OR^a^	Observed OR (95% CI)
0–1	0.37	0.39 (0.27–0.57)
0–10	0.52	0.55 (0.49–0.61)
10–20	0.67	0.71 (0.64–0.79)
20–30	0.77	0.74 (0.66–0.82)
30–40	0.86	0.88 (0.80–0.98)
40–60	1.00, reference	1.00, reference
60–70	1.16	1.10 (0.99–1.21)
70–80	1.29	1.24 (1.13–1.37)
80–90	1.49	1.52 (1.38–1.67)
90–100	1.97	1.93 (1.76–2.12)
99–100	2.77	2.70 (2.15–3.40)
OR per decile of PRS		1.13 (1.12–1.14)
SD^a^	0.38	
*c*-statistics for PRS^b^		0.602
*c*-statistic improvement^b^		0.0386 (0.0259–0.0513)

^a^Predicted ORs were estimated based on the PRS distribution with the SD 0.38

^b^The *c*-statistics and the improvement of *c*-statistics due to the PRS over the traditional risk factors (including age at menarche, age at first live birth, waist-to-hip ratio, breast cancer family history, and prior benign breast disease [21]) were estimated from the Shanghai breast cancer Genome-Wide Association Study

*CI* confidence interval, *OR* odds ratio, *PRS* polygenic risk score, *SD* standard deviation

**Fig. 1:**
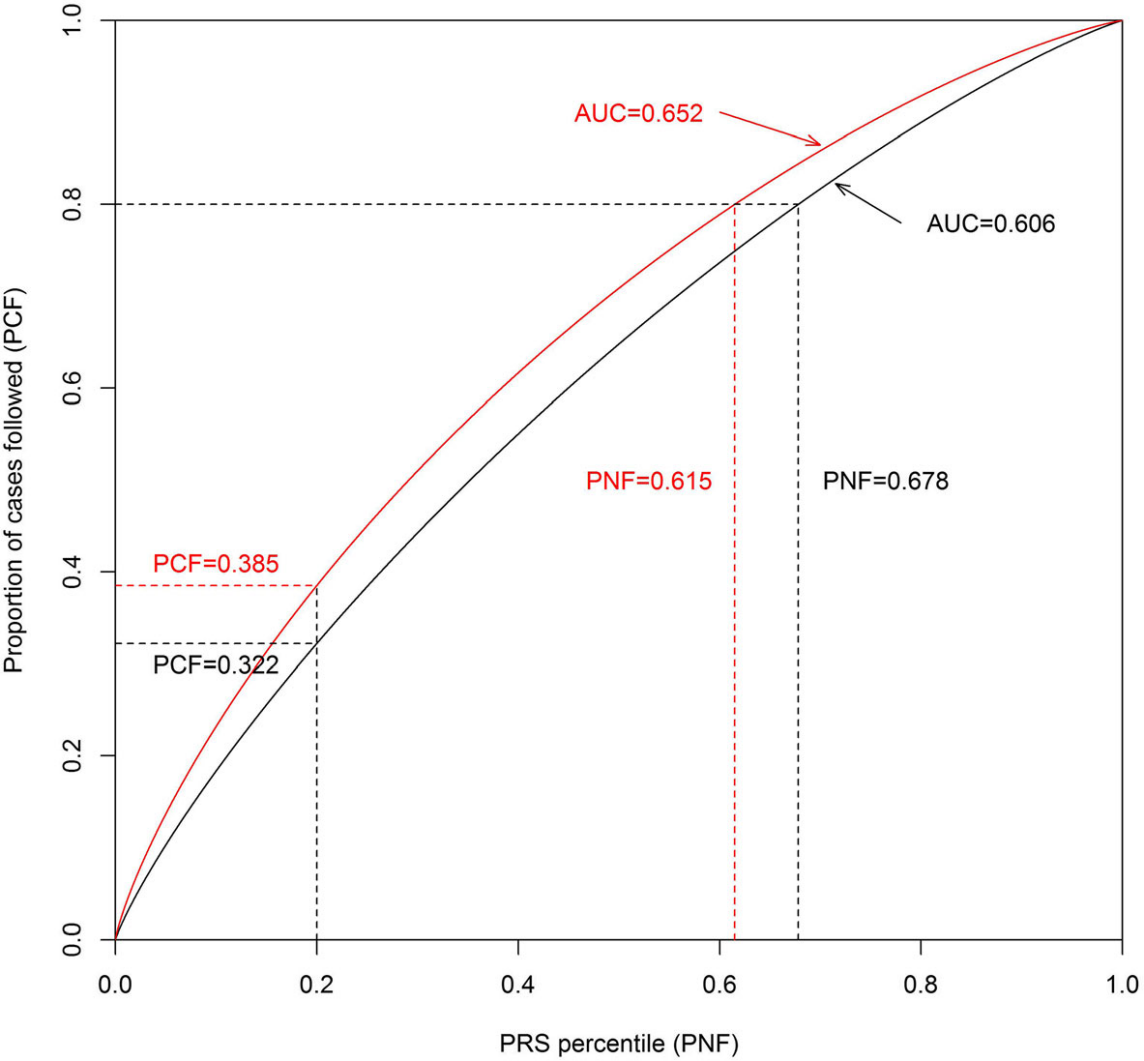
The proportion of cases followed (*PCF*) versus the polygenic risk score (*PRS*) percentile of proportion needed to follow-up (*PNF*). *AUC* area under the receiver operating characteristic curve

As mentioned earlier, the PCF measures the proportion of cases (*p*) which are included in the proportion *q* of individuals in the population at highest risk, while PNF assesses the proportion of the general population at highest risk (*q*) that one needs to follow in order that a proportion *p* of those destined to become cases will be followed. Given the SD of 0.38 for the PRS distribution, we estimated that approximately 2.6% of breast cancer cases in the general population would be found among those who were in the top 1% of PRS (PCF = 2.6% when PNF = 1%) (Table 2 and Fig. 1). In other words, to detect 80% of cases, 67.8% of the population needs to be screened (PNF = 67.8% when PCF = 80%). Given SD = 0.38, we estimated the AUC = 0.606, which is similar to the value of 0.602 estimated from logistic models using the data for 5152 Chinese women from the SGWAS. Figure 1 shows the AUC, which is also the area under a plot of PCF versus PNF as the risk threshold varies [18]. Based on the logistic models, the improvement in the AUC for the 44-SNP PRS to the breast cancer prediction model was 0.0386 (Table 1). This is greater than the AUC improvement (0.0328) for all of the traditional breast cancer risk factors combined from the same data (results not shown).

**Table 2:** Proportion of breast cancer cases followed versus the proportion of the general population at highest risk

PNF (%)	PCF (%)	
	PRS, SD = 0.38^a^	PRS, SD = 0.55^b^
1	2.6	3.8
5	10.3	13.7
10	18.4	23.2
20	32.2	38.5
30	44.3	51.0
40	55.0	61.7
50	64.8	70.9
60	73.7	78.9
70	81.7	85.9
80	88.9	91.8
90	95.2	96.6
95	97.9	98.6
99	99.7	99.8

^a^Observed SD of the PRS distribution in East Asian women

^b^Assumed SD of the PRS distribution, which corresponds to 30% of the heritability of breast cancer

*PCF* proportion of cases followed, *PNF* proportion needed to follow-up, *PRS* polygenic risk score, *SD* standard deviation

An estimate of 30% of the heritability of breast cancer, the total variability of propensity for breast cancer explained by genetic factors, was reported [23, 24], which corresponds to SD = 0.55 for the genetic variation. We present the AUC, PCF, and PNF for SD = 0.55 in Table 2 for comparison purposes. We estimated the AUC = 0.652 when SD = 0.55.

The absolute risk estimates for Shanghai Chinese and Korean women were compared (Table 3). Using the predicted OR estimates in Eq. (7), the estimated PAR (Eq. (8)) for breast cancer is 6.8% for the 44-SNP PRS. According to this PRS value, and using Eq. (6) and the age-specific breast cancer incidence and age-specific nonbreast cancer mortality for women in Shanghai in 2002 and 2003 [21] or in Korean women [22], the lifetime risk (age 20–80) of developing breast cancer by age 80 for the lowest 1% of the PRS was 1.35% for Chinese women in Shanghai and 1.31% for Korean women. The estimated risk for the highest 1% of the PRS was 10.06% for Chinese women and 9.81% for Korean women. For a 50-year-old woman with an average PRS value (40th–60th percentiles), the projected 10-year absolute risk of breast cancer is 1.03% for Chinese women and 1.05% for Korean women.

**Table 3:** Absolute risk estimated from the predicted OR, by the PRS percentiles

		Shanghai Chinese women		Korean women	
PRS (%)	Predicted OR	Lifetime risk (%)^a^	10-year risk (%)^b^	Lifetime risk (%)^a^	10-year risk (%)^b^
0–1	0.37	1.35	0.38	1.31	0.39
0–10	0.52	1.89	0.53	1.85	0.55
10–20	0.67	2.44	0.69	2.38	0.70
20–30	0.77	2.80	0.79	2.73	0.81
30–40	0.86	3.13	0.88	3.05	0.90
40–60	1.00	3.64	1.03	3.55	1.05
60–70	1.16	4.22	1.19	4.12	1.22
70–80	1.29	4.69	1.32	4.58	1.35
80–90	1.49	5.42	1.53	5.28	1.56
90–100	1.97	7.16	2.02	6.98	2.07
99–100	2.77	10.06	2.84	9.81	2.90

^a^Lifetime risk: the risk of developing breast cancer from age 20 to 80

^b^Ten-year risk: the risk of developing breast cancer from age 50 to 60

*OR* odds ratio, *PRS* polygenic risk score

As reported previously [13], we observed significant heterogeneity (*P* < 0.05) of the SNP–breast cancer association by breast cancer estrogen receptor (ER) status in multiple loci (Additional file 3: Table S3 and Additional file 4: Table S4). As a whole, for the PRS distribution under the multiplicative polygenic model (Eq. (3)), we observed an SD of the PRS of 0.39 for ER-positive breast cancer and 0.38 for ER-negative breast cancer.

Finally, we evaluated the interaction between the PRS and age and pairwise multiplicative SNP by SNP interaction; no significant results were observed.

## Discussion

In this study, we demonstrated the value of using common breast cancer variants, summarized as a 44-SNP PRS, to discriminate the breast cancer risk for women of East Asian ancestry. Compared with the recent report for women of European ancestry [15], we found that the PRS of common genetic variants had a smaller discriminative ability to identify high breast cancer risk in Asian women. The SD of the PRS distribution was 0.45 in European women, while the SD in this report among East Asian women is 0.38. There were 34 breast cancer loci identified previously in populations of European ancestry that were not associated with breast cancer risk in Asian women. In addition, previous studies found that the association of the PRS with ER-positive breast cancer was substantially stronger than the association with ER-negative breast cancer in women of European ancestry [25]. Mavaddat et al. [15] observed a striking difference in the SD of the PRS distribution by ER status (SD of 0.50 for ER-positive breast cancer and 0.38 for ER-negative breast cancer) in women of European ancestry. By comparison, a much less striking difference in the SD of the PRS distribution by ER status was observed (SD of 0.39 for ER-positive breast cancer and 0.38 for ER-negative breast cancer) in women of Asian ancestry (Additional file 3: Table S3 and Additional file 4: Table S4).

We reported previously the contribution of a genetic risk score derived from eight breast cancer-related SNPs in the prediction of breast cancer risk [21]. The 44-SNP PRS had greater discriminative ability than the eight-SNP PRS reported previously [21]. The AUC improvement of 0.0386 and SD = 0.38 for the 44-SNP PRS were substantially greater than the AUC improvement of 0.0117 and SD = 0.21 for the previous eight-SNP PRS. Previously we estimated that 37.7% of breast cancer cases in the general population would be found among women in the top 30% of the eight-SNP PRS values. Based on the 44-SNP PRS, we would expect to find 44.3% of breast cancer cases among those women, a moderate improvement for targeting women with a high risk of breast cancer for screening. If all genetic effects, estimated according to 30% of heritability of breast cancer [23, 24], were taken into account, we would find 51% of breast cancer cases among women in the top 30% of genetic risk (Table 2).

A limitation of this study is that this analysis included original studies that identified several new genetic variants among women of Asian ancestry [8–12], which raised an overfitting concern for the prediction model. If those SNPs were excluded from the PRS, then the SD of the PRS would be slightly decreased to 0.37 from 0.38, and the AUC would be slightly decreased to 0.603 from 0.606. On the contrary, it can be anticipated that the discriminative ability of breast cancer risk prediction based on genetic factors will further increase as more studies are conducted and more genetic variants, common or rare, are identified in East Asian women. In this report, there were several loci whose association with breast cancer risk in Asian women were not significant but were within the 95% CI of the association for European populations (Additional file 2: Tables S2). If those loci were included in the PRS, then the SD of the PRS would be slightly increased to 0.39 from 0.38, and the AUC would be slightly increased to 0.609 from 0.606. However, even when all genetic factors are taken into account (AUC = 0.652), the improvement in discrimination quality would still not be sufficient to be considered meaningful for clinical application. In order to increase discriminatory accuracy, other strong predictors, such as mammographic density and biopsy features, need to be included.

## Conclusions

We have shown that known common genetic variants are important predictors for breast cancer risk, and using a 44-SNP PRS could help discriminate breast cancer risk in women of East Asian ancestry, although the discriminatory ability is not sufficient for clinical application.

## Additional files

Additional file 1: is Table S1 presenting participating studies in this analysis. (PDF 79 kb)
Additional file 2: is Table S2 presenting the association between selected SNPs and breast cancer risk in East Asian women. (PDF 146 kb)
Additional file 3: is Table S3 presenting the association between selected SNPs and ER-positive breast cancer risk in East Asian women. (PDF 134 kb)
Additional file 4: is Table S4 presenting the association between selected SNPs and ER-negative breast cancer risk in East Asian women. (PDF 137 kb)
Additional file 5: is Table S5 presenting ethics committees that approved participating studies. (PDF 59 kb)

## Abbreviations

AUC: Area under the receiver operating characteristic curve
BCAC: Breast Cancer Association Consortium
CI: Confidence interval
COGS: Collaborative Oncological Gene–environment Study
ER: Estrogen receptor
GWAS: Genome-wide association study
OR: Odds ratio
PAR: Population attributable risk
PCF: Proportion of cases followed
PNF: Proportion needed to follow-up
PRS: Polygenic risk score
QC: Quality control
SBCS: Shanghai Breast Cancer Study
SBCSS: Shanghai Breast Cancer Survival Study
SD: Standard deviation
SGWAS: Shanghai Genome-wide Association Studies
SNP: Single nucleotide polymorphism
SWHS: Shanghai Women’s Health Study
