# Supplementary material for: Prediction of breast cancer risk based on common genetic variants in women of East Asian ancestry
Source: Breast Cancer Res. 2016 Dec 8;18:124. doi: 10.1186/s13058-016-0786-1 (PMC5146840; doi:10.1186/s13058-016-0786-1)
Supplement: Additional file 1: — is Table S1 presenting participating studies in this analysis. (PDF 79 kb) [file 13058_2016_786_MOESM1_ESM.pdf]

Table S1. Participating studies in this analysis

Study	Population	Case	Control	Total
**BCAC**				
ACP	Thai	423	636	1059
HERPACC	Japanese	694	1376	2070
LAABC	Japanese/Chinese	812	990	1802
MYBRCA	Chinese/Malaysian	770	610	1380
SBCGS	Chinese	848	892	1740
SEBCS	Korean	1162	1129	2291
SGBCC	Chinese/Malaysian	533	502	1035
TBCS	Thai	138	253	391
TWBCS	Chinese	889	236	1125
**Shanghai**				
SGWAS	Chinese	2867	2285	5152
SGWAS_stage2	Chinese	2769	2753	5522
**Total**		11 905	11 662	23 567
