# Supplementary material for: Prediction of breast cancer risk based on common genetic variants in women of East Asian ancestry
Source: Breast Cancer Res. 2016 Dec 8;18:124. doi: 10.1186/s13058-016-0786-1 (PMC5146840; doi:10.1186/s13058-016-0786-1)
Supplement: Additional file 2: — is Table S2 presenting the association between selected SNPs and breast cancer risk in East Asian women. (PDF 146 kb) [file 13058_2016_786_MOESM2_ESM.pdf]

**Table S2. The Association between selected SNPs and breast cancer risk in East Asian women.**

| | | | | | | East Asian women | | | | | European women |
|---|---|---|---|---|---|---|---|---|---|---|---|
| Chr | Position | SNP | gene | EA[a] | OA[a] | EAF[a] | Number | beta | se | One-sided P | beta (95 % CI)[b] |
| 44 SNPs used for the construction of the polygenic genetic score | | | | | | | | | | | |
| 1 | 10566215 | rs616488 | PEX14 | A | G | 0.70 | 23564 | 0.070 | 0.020 | 2.96E-04 | 0.058(0.038-0.077) |
| 1 | 121280613 | rs11249433 | EMBP1 | G | A | 0.08 | 23566 | 0.088 | 0.052 | 4.51E-02 | 0.093(0.073-0.112) |
| 1 | 203766331 | rs4951011 | ZC3H11A | G | A | 0.31 | 23567 | 0.056 | 0.021 | 3.47E-03 | 0.034(0.006-0.063) |
| 2 | 19320803 | rs12710696 | MIR4757 | T | C | 0.33 | 18044 | 0.048 | 0.023 | 1.91E-02 | 0.039(0.019-0.058) |
| 2 | 121245122 | rs4849887 | LOC84931 | C | T | 0.80 | 23567 | 0.065 | 0.024 | 2.97E-03 | 0.100(0.068-0.132) |
| 2 | 202143928 | rs10931936 | CASP8 | T | C | 0.29 | 18045 | 0.053 | 0.024 | 1.30E-02 | 0.042(0.021-0.062) |
| 2 | 217905832 | rs13387042 | TNP1 | A | G | 0.12 | 23567 | 0.056 | 0.029 | 2.87E-02 | 0.132(0.114-0.151) |
| 2 | 218296508 | rs16857609 | DIRC3 | T | C | 0.60 | 23563 | 0.084 | 0.019 | 6.80E-06 | 0.079(0.058-0.100) |
| 3 | 27416013 | rs4973768 | SLC4A7 | T | C | 0.21 | 23564 | 0.107 | 0.023 | 1.79E-06 | 0.094(0.075-0.113) |
| 3 | 30682939 | rs12493607 | GFBR2 | C | G | 0.67 | 18044 | 0.050 | 0.023 | 1.62E-02 | 0.054(0.035-0.073) |
| 4 | 175846426 | rs6828523 | ADAM29 | C | A | 0.75 | 18044 | 0.058 | 0.025 | 9.25E-03 | 0.108(0.079-0.137) |
| 5 | 1279790 | rs10069690 | TERT | T | C | 0.20 | 23562 | 0.065 | 0.026 | 6.40E-03 | 0.059(0.038-0.080) |
| 5 | 44706498 | rs10941679 | MRPS30 | G | A | 0.50 | 18045 | 0.067 | 0.022 | 1.26E-03 | 0.120(0.099-0.141) |
| 5 | 56031884 | rs889312 | MAP3K1 | C | A | 0.55 | 23564 | 0.038 | 0.019 | 2.22E-02 | 0.115(0.095-0.136) |
| 5 | 90732225 | rs10474352 | LOC100129716 | C | T | 0.55 | 23567 | 0.077 | 0.020 | 5.60E-05 | 0.058(0.027-0.088) |
| 5 | 158244083 | rs1432679 | EBF1 | C | T | 0.65 | 23567 | 0.068 | 0.020 | 3.26E-04 | 0.070(0.051-0.089) |
| 6 | 149608874 | rs9485372 | TAB2 | G | A | 0.58 | 18045 | 0.128 | 0.022 | 3.96E-09 | 0.048(0.024-0.073) |
| 6 | 151948366 | rs2046210 | C6orf97 | A | G | 0.38 | 23567 | 0.237 | 0.020 | 0.00E+00 | 0.078(0.058-0.097) |
| 8 | 29509616 | rs9693444 | C8orf75 | A | C | 0.29 | 23566 | 0.074 | 0.021 | 1.80E-04 | 0.068(0.048-0.087) |
| 8 | 76230301 | rs6472903 | HNF4G | T | G | 0.96 | 23567 | 0.118 | 0.049 | 7.80E-03 | 0.095(0.070-0.120) |
| 8 | 128387852 | rs1562430 | POU5F1B | T | C | 0.83 | 23567 | 0.067 | 0.025 | 3.85E-03 | 0.106(0.087-0.125) |
| 9 | 22062134 | rs1011970 | CDKN2B-AS1 | T | G | 0.09 | 23566 | 0.063 | 0.033 | 2.66E-02 | 0.054(0.029-0.079) |
| 9 | 110306115 | rs10759243 | 9q31.2 | A | C | 0.44 | 18045 | 0.057 | 0.022 | 4.36E-03 | 0.048(0.028-0.069) |
| 10 | 64251977 | rs10822013 | ZNF365 | T | C | 0.50 | 23567 | 0.075 | 0.019 | 3.48E-05 | 0.063(0.044-0.082) |
| 10 | 80841148 | rs704010 | ZMIZ1 | T | C | 0.32 | 23564 | 0.063 | 0.020 | 9.35E-04 | 0.081(0.062-0.100) |

| | | | | | | | | | | | |
|---|---|---|---|---|---|---|---|---|---|---|---|
| 10 | 123093901 | rs11199914 | 10q26.12 | C | T | 0.62 | 18045 | 0.042 | 0.022 | 3.07E-02 | 0.051(0.031-0.071) |
| 10 | 123337335 | rs2981579 | FGFR2 | A | G | 0.46 | 23567 | 0.151 | 0.019 | 1.34E-15 | 0.236(0.217-0.255) |
| 11 | 1941946 | rs909116 | TNNT3 | T | C | 0.39 | 23555 | 0.075 | 0.020 | 1.09E-04 | 0.070(0.051-0.089) |
| 11 | 69328764 | rs614367 | CCND1 | T | C | 0.01 | 23565 | 0.249 | 0.094 | 4.02E-03 | 0.194(0.169-0.220) |
| 11 | 129473690 | rs7107217 | BARX2 | C | A | 0.37 | 18040 | 0.095 | 0.023 | 1.25E-05 | 0.045(0.026-0.063) |
| 12 | 14413931 | rs12422552 | ATF7IP | C | G | 0.29 | 18043 | 0.054 | 0.024 | 1.36E-02 | 0.042(0.021-0.063) |
| 12 | 28155080 | rs10771399 | PTHLH | A | G | 0.82 | 23567 | 0.112 | 0.024 | 2.09E-06 | 0.159(0.130-0.189) |
| 12 | 96027759 | rs17356907 | NTN4 | A | G | 0.76 | 23567 | 0.063 | 0.022 | 2.21E-03 | 0.093(0.073-0.114) |
| 12 | 115836522 | rs1292011 | MED13L | A | G | 0.75 | 23566 | 0.117 | 0.022 | 4.95E-08 | 0.084(0.065-0.103) |
| 14 | 37132769 | rs2236007 | PAX9 | G | A | 0.72 | 23561 | 0.071 | 0.021 | 4.22E-04 | 0.076(0.053-0.099) |
| 14 | 91841069 | rs941764 | CCDC88C | G | A | 0.15 | 23565 | 0.062 | 0.027 | 9.55E-03 | 0.063(0.043-0.082) |
| 15 | 91512067 | rs2290203 | PRC1 | G | A | 0.51 | 18045 | 0.081 | 0.022 | 8.15E-05 | 0.039(0.015-0.062) |
| 16 | 52586341 | rs3803662 | LOC643714 | A | G | 0.63 | 23565 | 0.129 | 0.020 | 2.64E-11 | 0.215(0.194-0.236) |
| 16 | 52599188 | rs4784227 | LOC643714 | T | C | 0.27 | 23567 | 0.209 | 0.022 | 0.00E+00 | 0.227(0.206-0.249) |
| 16 | 53855291 | rs11075995 | FTO | A | T | 0.31 | 18044 | 0.071 | 0.024 | 1.19E-03 | 0.043(0.021-0.065) |
| 18 | 24337424 | rs527616 | LOC728606 | G | C | 0.71 | 23566 | 0.042 | 0.021 | 2.27E-02 | 0.054(0.035-0.073) |
| 19 | 17394124 | rs2363956 | ANKLE1 | T | G | 0.68 | 23562 | 0.057 | 0.021 | 2.88E-03 | 0.027(0.008-0.045) |
| 19 | 18571141 | rs4808801 | ELL | A | G | 0.75 | 23561 | 0.055 | 0.022 | 5.65E-03 | 0.078(0.058-0.098) |
| 22 | 39358037 | rs12628403 | APOBEC3A | C | A | 0.34 | 23561 | 0.108 | 0.024 | 4.56E-06 | 0.051(0.011-0.091) |
| Other SNPs | | | | | | | | | | | |
| 1 | 114448389 | rs11552449 | DCLRE1B | T | C | 0.60 | 23566 | 0.018 | 0.020 | 1.88E-01 | 0.066(0.041-0.091) |
| 1 | 202187176 | rs6678914 | LGR6 | G | A | 0.77 | 18044 | 0.012 | 0.026 | 3.19E-01 | 0.009(-0.010-0.028) |
| 1 | 204518842 | rs4245739 | MDM4 | C | A | 0.05 | 18043 | -0.037 | 0.051 | 7.67E-01 | 0.033(0.012-0.054) |
| 2 | 172972971 | rs2016394 | METAP1D | G | A | 0.79 | 18045 | 0.016 | 0.028 | 2.87E-01 | 0.044(0.025-0.062) |
| 2 | 174212894 | rs1550623 | CDCA7 | A | G | 0.98 | 18040 | 0.140 | 0.092 | 6.35E-02 | 0.060(0.034-0.086) |
| 2 | 202149589 | rs1045485 | CASP8 | G | C | 1.00 | 23565 | -0.178 | 0.303 | 7.21E-01 | 0.030(0.002-0.058) |
| 3 | 4742276 | rs6762644 | ITPR1/EGOT | G | A | 0.09 | 23566 | 0.039 | 0.034 | 1.22E-01 | 0.066(0.047-0.085) |
| 4 | 106084778 | rs9790517 | TET2 | T | C | 0.60 | 18043 | 0.010 | 0.022 | 3.24E-01 | 0.057(0.035-0.080) |
| 5 | 44899885 | rs9790879 | MRPS30 | T | C | 0.44 | 18042 | -0.041 | 0.022 | 9.69E-01 | 0.085(0.066-0.104) |
| 5 | 58184061 | rs10472076 | RAB3C | C | T | 0.26 | 23566 | 0.011 | 0.022 | 2.99E-01 | 0.043(0.023-0.062) |
| 5 | 58337481 | rs1353747 | PDE4D | T | G | 1.00 | 18045 | 0.204 | 0.187 | 1.38E-01 | 0.074(0.042-0.106) |

| | | | | | | | | | | | |
|---|---|---|---|---|---|---|---|---|---|---|---|
| 6 | 1318878 | rs11242675 | FOXQ1 | T | C | 0.45 | 23566 | -0.019 | 0.019 | 8.47E-01 | 0.038(0.019-0.058) |
| 6 | 13722523 | rs204247 | RANBP9 | G | A | 0.60 | 23566 | 0.023 | 0.019 | 1.16E-01 | 0.047(0.028-0.066) |
| 6 | 82193109 | rs17530068 | FAM46A | C | T | 0.22 | 23567 | 0.031 | 0.023 | 8.35E-02 | 0.054(0.032-0.076) |
| 6 | 151914113 | rs3757318 | C6orf97 | A | G | 0.28 | 23566 | 0.167 | 0.022 | 4.11E-15 | 0.146(0.110-0.181) |
| 7 | 144074929 | rs720475 | ARHGEF5/NOBO | G | A | 0.96 | 18045 | 0.025 | 0.059 | 3.36E-01 | 0.059(0.037-0.081) |
| 8 | 76417937 | rs2943559 | HNF4G | G | A | 0.09 | 18045 | -0.038 | 0.039 | 8.38E-01 | 0.128(0.093-0.163) |
| 8 | 128355618 | rs13281615 | POU5F1B | G | A | 0.52 | 23429 | 0.021 | 0.019 | 1.38E-01 | 0.093(0.074-0.112) |
| 8 | 129194641 | rs11780156 | MIR1208 | T | C | 0.21 | 18045 | -0.026 | 0.026 | 8.38E-01 | 0.068(0.043-0.093) |
| 9 | 110888478 | rs865686 | 9q31 | T | G | 0.93 | 23567 | 0.056 | 0.037 | 6.65E-02 | 0.103(0.084-0.123) |
| 10 | 5886734 | rs2380205 | ANKRD16 | C | T | 0.88 | 18042 | 0.016 | 0.033 | 3.14E-01 | 0.013(-0.006-0.032) |
| 10 | 22032942 | rs7072776 | MLLT10/DNAJ | A | G | 0.06 | 23567 | 0.002 | 0.045 | 4.81E-01 | 0.062(0.042-0.083) |
| 10 | 22315843 | rs11814448 | DNAJC1 | C | A | 0.01 | 23567 | 0.064 | 0.096 | 2.53E-01 | 0.238(0.174-0.301) |
| 10 | 64278682 | rs10995190 | ZNF365 | G | A | 0.98 | 23567 | -0.017 | 0.065 | 6.02E-01 | 0.149(0.123-0.175) |
| 10 | 114773927 | rs7904519 | TCF7L2 | G | A | 0.07 | 23566 | 0.060 | 0.052 | 1.24E-01 | 0.056(0.037-0.075) |
| 10 | 123352317 | rs2981582 | FGFR2 | A | G | 0.33 | 23565 | 0.115 | 0.020 | 3.87E-09 | 0.227(0.208-0.246) |
| 11 | 1909006 | rs3817198 | LSP1 | C | T | 0.14 | 23565 | 0.084 | 0.027 | 1.05E-03 | 0.066(0.046-0.087) |
| 11 | 65583066 | rs3903072 | OVOL1 | G | T | 0.79 | 18045 | 0.046 | 0.027 | 4.26E-02 | 0.054(0.035-0.073) |
| 11 | 129461171 | rs11820646 | BARX2 | C | T | 0.54 | 23565 | 0.041 | 0.019 | 1.78E-02 | 0.051(0.032-0.070) |
| 13 | 32972626 | rs11571833 | BRCA2 | T | A | 0.00 | 23566 | 0.496 | 0.538 | 1.79E-01 | 0.246(0.149-0.343) |
| 14 | 68660428 | rs2588809 | RAD51L1 | T | C | 0.03 | 23567 | 0.053 | 0.062 | 1.94E-01 | 0.075(0.050-0.101) |
| 14 | 69034682 | rs999737 | RAD51B | C | T | 1.00 | 23566 | -0.081 | 0.154 | 7.01E-01 | 0.084(0.061-0.106) |
| 14 | 69039588 | rs8009944 | RAD51B | C | A | 0.73 | 18044 | 0.030 | 0.025 | 1.10E-01 | 0.035(0.014-0.056) |
| 16 | 52548037 | rs12443621 | TOX3 | G | A | 0.57 | 18045 | 0.013 | 0.022 | 2.84E-01 | 0.120(0.101-0.139) |
| 16 | 53813367 | rs17817449 | MIR1972 | T | G | 0.86 | 23566 | 0.044 | 0.027 | 5.30E-02 | 0.072(0.053-0.091) |
| 16 | 80650805 | rs13329835 | CDYL2 | G | A | 0.05 | 23567 | 0.058 | 0.042 | 8.55E-02 | 0.078(0.056-0.101) |
| 17 | 53056471 | rs6504950 | STXBP4/COX1 | G | A | 0.91 | 18045 | -0.010 | 0.038 | 6.00E-01 | 0.062(0.041-0.083) |
| 18 | 24570667 | rs1436904 | CHST9 | T | G | 0.54 | 23564 | 0.029 | 0.019 | 6.30E-02 | 0.049(0.030-0.068) |
| 19 | 17389704 | rs8170 | BABAM1 | A | G | 0.01 | 23566 | 0.121 | 0.170 | 2.38E-01 | 0.040(0.016-0.063) |
| 19 | 44286513 | rs3760982 | KCNN4/ZNF28 | A | G | 0.16 | 23567 | -0.017 | 0.026 | 7.45E-01 | 0.053(0.034-0.071) |
| 20 | 32588095 | rs2284378 | RALY | T | C | 0.18 | 18045 | -0.015 | 0.028 | 7.02E-01 | 0.017(-0.003-0.037) |
| 21 | 16520832 | rs2823093 | NRIP1 | G | A | 0.96 | 18045 | 0.068 | 0.055 | 1.09E-01 | 0.075(0.054-0.097) |

| | | | | | | | | | | | |
|---|---|---|---|---|---|---|---|---|---|---|---|
| 22 | 29621477 | rs132390 | EMID1/RHBDD3 | C | T | 0.00 | 18045 | 0.038 | 0.271 | 4.44E-01 | 0.123(0.074-0.172) |
| 22 | 40876234 | rs6001930 | MKL1 | C | T | 0.25 | 23566 | 0.022 | 0.022 | 1.55E-01 | 0.117(0.088-0.147) |

[a] Abbreviations: EA, effect allele; OA, other allele; EAF, effect allele frequency.

[b] The association, beta and their 95% confidence intervals, between the selected SNPs and breast cancer risk, estimated from 91,767 women of European ancestry participating in the Breast Cancer Association Consortium [Mavaddat N, Pharoah P, Michailidou K. Prediction of breast cancer risk based on profiling with common genetic variants. *J. Natl. Cancer Inst.* 2015;107(5) djv036].
