# Supplementary material for: Prediction of breast cancer risk based on common genetic variants in women of East Asian ancestry
Source: Breast Cancer Res. 2016 Dec 8;18:124. doi: 10.1186/s13058-016-0786-1 (PMC5146840; doi:10.1186/s13058-016-0786-1)
Supplement: Additional file 3: — is Table S3 presenting the association between selected SNPs and ER-positive breast cancer risk in East Asian women. (PDF 134 kb) [file 13058_2016_786_MOESM3_ESM.pdf]

Table S3. The association between selected SNPs and ER positive breast cancer risk in East Asian women.

Chr	Position	SNP	Gene	EA[a]	OA[a]	EAF[a]	Among ER positive cases and all controls					
							Number	beta	OR	se	one-sided p	Homo_p[b]
44 SNPs used for the construction of the polygenic genetic score												
1	10566215	rs616488	PEX14	A	G	0.70	18306	0.0614	1.06	0.0241	5.50E-03	1.97E-01
1	121280613	rs11249433	EMBP1	G	A	0.08	18308	0.1387	1.15	0.0611	1.17E-02	3.61E-01
1	203766331	rs4951011	ZC3H11A	G	A	0.31	18309	0.0446	1.05	0.0245	3.45E-02	3.83E-01
2	19320803	rs12710696	MIR4757	T	C	0.33	13837	0.0447	1.05	0.0276	5.30E-02	7.17E-01
2	121245122	rs4849887	LOC84931	C	T	0.80	18309	0.0608	1.06	0.0281	1.52E-02	5.85E-01
2	202143928	rs10931936	CASP8	T	C	0.29	13838	0.0724	1.08	0.0282	5.15E-03	6.60E-01
2	217905832	rs13387042	TNP1	A	G	0.12	18309	0.0738	1.08	0.0346	1.65E-02	1.84E-01
2	218296508	rs16857609	DIRC3	T	C	0.60	18305	0.0930	1.10	0.0228	2.19E-05	9.02E-01
3	27416013	rs4973768	SLC4A7	T	C	0.21	18306	0.1400	1.15	0.0273	1.43E-07	1.37E-01
3	30682939	rs12493607	GFBR2	C	G	0.67	13837	0.0555	1.06	0.0280	2.37E-02	5.19E-01
4	175846426	rs6828523	ADAM29	C	A	0.75	13837	0.0786	1.08	0.0298	4.19E-03	1.89E-01
5	1279790	rs10069690	TERT	T	C	0.20	18304	0.0320	1.03	0.0310	1.51E-01	2.22E-02
5	44706498	rs10941679	MRPS30	G	A	0.50	13838	0.0862	1.09	0.0266	6.10E-04	2.10E-02
5	56031884	rs889312	MAP3K1	C	A	0.55	18306	0.0502	1.05	0.0225	1.29E-02	2.70E-01
5	90732225	rs10474352	LOC100129716	C	T	0.55	18309	0.0736	1.08	0.0235	8.85E-04	5.98E-01
5	158244083	rs1432679	EBF1	C	T	0.65	18309	0.0651	1.07	0.0236	2.96E-03	5.68E-01
6	149608874	rs9485372	TAB2	G	A	0.58	13838	0.1073	1.11	0.0266	2.72E-05	5.13E-01
6	151948366	rs2046210	C6orf97	A	G	0.38	18309	0.2052	1.23	0.0231	0.00E+00	7.67E-03
8	29509616	rs9693444	C8orf75	A	C	0.29	18308	0.0625	1.06	0.0245	5.35E-03	8.23E-01
8	76230301	rs6472903	HNF4G	T	G	0.96	18309	0.1416	1.15	0.0583	7.60E-03	5.71E-01
8	128387852	rs1562430	POU5F1B	T	C	0.83	18309	0.0575	1.06	0.0297	2.64E-02	8.73E-01
9	22062134	rs1011970	CDKN2B-AS1	T	G	0.09	18308	0.0157	1.02	0.0390	3.44E-01	1.21E-01
9	110306115	rs10759243	9q31.2	A	C	0.44	13838	0.0568	1.06	0.0260	1.44E-02	6.82E-01
10	64251977	rs10822013	ZNF365	T	C	0.50	18309	0.0707	1.07	0.0222	7.45E-04	8.71E-01
10	80841148	rs704010	ZMIZ1	T	C	0.32	18306	0.0665	1.07	0.0237	2.57E-03	9.29E-01
10	123093901	rs11199914	10q26.12	C	T	0.62	13838	0.0737	1.08	0.0267	2.95E-03	9.52E-02
10	123337335	rs2981579	FGFR2	A	G	0.46	18309	0.2006	1.22	0.0226	0.00E+00	1.42E-03
11	1941946	rs909116	TNNT3	T	C	0.39	18297	0.0708	1.07	0.0241	1.63E-03	9.63E-01
11	69328764	rs614367	CCND1	T	C	0.01	18307	0.3645	1.44	0.1161	8.50E-04	6.42E-02

11	129473690	rs7107217	BARX2	C	A	0.37	13833	0.0986	1.10	0.0269	1.26E-04	8.63E-01
12	14413931	rs12422552	ATF7IP	C	G	0.29	13836	0.0881	1.09	0.0292	1.27E-03	3.76E-02
12	28155080	rs10771399	PTHLH	A	G	0.82	18309	0.0871	1.09	0.0287	1.20E-03	3.40E-01
12	96027759	rs17356907	NTN4	A	G	0.76	18309	0.0586	1.06	0.0263	1.29E-02	7.62E-01
12	115836522	rs1292011	MED13L	A	G	0.75	18308	0.1062	1.11	0.0259	2.13E-05	3.64E-01
14	37132769	rs2236007	PAX9	G	A	0.72	18303	0.0845	1.09	0.0251	3.86E-04	6.61E-01
14	91841069	rs941764	CCDC88C	G	A	0.15	18307	0.0723	1.07	0.0315	1.08E-02	7.13E-01
15	91512067	rs2290203	PRC1	G	A	0.51	13838	0.0976	1.10	0.0258	7.70E-05	1.15E-01
16	52586341	rs3803662	LOC643714	A	G	0.63	18307	0.1516	1.16	0.0234	4.95E-11	2.12E-01
16	52599188	rs4784227	LOC643714	T	C	0.27	18309	0.2455	1.28	0.0252	0.00E+00	2.17E-02
16	53855291	rs11075995	FTO	A	T	0.31	13837	0.0736	1.08	0.0280	4.25E-03	4.87E-01
18	24337424	rs527616	LOC728606	G	C	0.71	18308	0.0622	1.06	0.0245	5.65E-03	1.39E-01
19	17394124	rs2363956	ANKLE1	T	G	0.68	18304	0.0306	1.03	0.0243	1.04E-01	3.03E-02
19	18571141	rs4808801	ELL	A	G	0.75	18303	0.0457	1.05	0.0257	3.77E-02	6.19E-01
22	39358037	rs12628403	APOBEC3A	C	A	0.34	18303	0.1072	1.11	0.0285	8.55E-05	9.77E-01
Other SNPs												
1	114448389	rs11552449	DCLRE1B	T	C	0.60	18308	0.0152	1.02	0.0238	2.62E-01	6.84E-01
1	202187176	rs6678914	LGR6	G	A	0.77	13837	-0.0061	0.99	0.0307	5.79E-01	3.26E-01
1	204518842	rs4245739	MDM4	C	A	0.05	13836	-0.0966	0.91	0.0631	9.37E-01	4.19E-01
2	172972971	rs2016394	METAP1D	G	A	0.79	13838	0.0310	1.03	0.0336	1.78E-01	3.89E-01
2	174212894	rs1550623	CDCA7	A	G	0.98	13833	0.1980	1.22	0.1152	4.29E-02	7.15E-01
2	202149589	rs1045485	CASP8	G	C	1.00	18307	-0.3171	0.73	0.3570	8.13E-01	9.94E-01
3	4742276	rs6762644	ITPR1/EGOT	G	A	0.09	18308	0.0535	1.05	0.0398	8.95E-02	7.30E-01
4	106084778	rs9790517	TET2	T	C	0.60	13836	-0.0029	1.00	0.0265	5.43E-01	3.65E-01
5	44899885	rs9790879	MRPS30	T	C	0.44	13835	-0.0519	0.95	0.0263	9.76E-01	1.28E-01
5	58184061	rs10472076	RAB3C	C	T	0.26	18308	0.0233	1.02	0.0254	1.80E-01	3.21E-01
5	58337481	rs1353747	PDE4D	T	G	1.00	13838	0.1516	1.16	0.2386	2.63E-01	7.00E-01
6	1318878	rs11242675	FOXQ1	T	C	0.45	18308	-0.0037	1.00	0.0224	5.66E-01	1.85E-01
6	13722523	rs204247	RANBP9	G	A	0.60	18308	0.0513	1.05	0.0228	1.21E-02	2.31E-02
6	82193109	rs17530068	FAM46A	C	T	0.22	18309	0.0056	1.01	0.0270	4.19E-01	1.77E-01
6	151914113	rs3757318	C6orf97	A	G	0.28	18308	0.1569	1.17	0.0253	3.03E-10	3.99E-01
7	144074929	rs720475	ARHGEF5/NOBO	G	A	0.96	13838	0.0638	1.07	0.0734	1.93E-01	1.67E-01
8	76417937	rs2943559	HNF4G	G	A	0.09	13838	-0.0192	0.98	0.0462	6.62E-01	5.20E-01
8	128355618	rs13281615	POU5F1B	G	A	0.52	18171	0.0187	1.02	0.0226	2.04E-01	9.36E-01

8	129194641	rs11780156	MIR1208	T	C	0.21	13838	-0.0279	0.97	0.0315	8.13E-01	9.24E-01
9	110888478	rs865686	9q31	T	G	0.93	18309	0.0536	1.06	0.0446	1.15E-01	6.34E-01
10	5886734	rs2380205	ANKRD16	C	T	0.88	13835	0.0137	1.01	0.0403	3.67E-01	4.49E-01
10	22032942	rs7072776	MLLT10/DNAJ	A	G	0.06	18309	-0.0210	0.98	0.0548	6.49E-01	8.60E-01
10	22315843	rs11814448	DNAJC1	C	A	0.01	18309	0.1107	1.12	0.1136	1.65E-01	6.25E-01
10	64278682	rs10995190	ZNF365	G	A	0.98	18309	0.0639	1.07	0.0785	2.08E-01	3.33E-01
10	114773927	rs7904519	TCF7L2	G	A	0.07	18308	0.0860	1.09	0.0633	8.75E-02	6.68E-01
10	123352317	rs2981582	FGFR2	A	G	0.33	18307	0.1714	1.19	0.0235	1.58E-13	9.21E-04
11	1909006	rs3817198	LSP1	C	T	0.14	18307	0.0822	1.09	0.0321	5.30E-03	6.72E-01
11	65583066	rs3903072	OVOL1	G	T	0.79	13838	0.0366	1.04	0.0325	1.31E-01	7.64E-01
11	129461171	rs11820646	BARX2	C	T	0.54	18307	0.0454	1.05	0.0228	2.34E-02	9.12E-01
13	32972626	rs11571833	BRCA2	T	A	0.00	18308	0.7510	2.12	0.5573	8.90E-02	7.81E-01
14	68660428	rs2588809	RAD51L1	T	C	0.03	18309	0.0307	1.03	0.0763	3.44E-01	7.05E-01
14	69034682	rs999737	RAD51B	C	T	1.00	18308	-0.1452	0.86	0.1871	7.81E-01	9.11E-01
14	69039588	rs8009944	RAD51B	C	A	0.73	13837	0.0461	1.05	0.0298	6.10E-02	5.08E-01
16	52548037	rs12443621	TOX3	G	A	0.57	13838	-0.0035	1.00	0.0262	5.54E-01	3.85E-02
16	53813367	rs17817449	MIR1972	T	G	0.86	18308	0.0357	1.04	0.0322	1.34E-01	6.91E-01
16	80650805	rs13329835	CDYL2	G	A	0.05	18309	0.0745	1.08	0.0492	6.50E-02	7.24E-01
17	53056471	rs6504950	STXBP4/COX1	G	A	0.91	13838	0.0168	1.02	0.0462	3.59E-01	9.44E-01
18	24570667	rs1436904	CHST9	T	G	0.54	18306	0.0285	1.03	0.0224	1.01E-01	9.14E-01
19	17389704	rs8170	BABAM1	A	G	0.01	18308	0.3214	1.38	0.2050	5.85E-02	4.91E-01
19	44286513	rs3760982	KCNN4/ZNF28	A	G	0.16	18309	0.0060	1.01	0.0310	4.23E-01	9.37E-01
20	32588095	rs2284378	RALY	T	C	0.18	13838	-0.0427	0.96	0.0336	8.99E-01	5.04E-01
21	16520832	rs2823093	NRIP1	G	A	0.96	13838	0.1427	1.15	0.0686	1.87E-02	1.37E-01
22	29621477	rs132390	EMID1/RHBDD3	C	T	0.00	13838	0.1692	1.18	0.3319	3.05E-01	5.56E-01
22	40876234	rs6001930	MKL1	C	T	0.25	18308	0.0545	1.06	0.0252	1.54E-02	2.09E-02

[a]Abbreviations: EA, effect allele; OA, other allele; EAF, effect allele frequency.

[b]Homo_p: p values for homogeneity tests between ER positive and ER negative breast cancer cases.
