# Supplementary material for: Prediction of breast cancer risk based on common genetic variants in women of East Asian ancestry
Source: Breast Cancer Res. 2016 Dec 8;18:124. doi: 10.1186/s13058-016-0786-1 (PMC5146840; doi:10.1186/s13058-016-0786-1)
Supplement: Additional file 4: — is Table S4 presenting the association between selected SNPs and ER-negative breast cancer risk in East Asian women. (PDF 137 kb) [file 13058_2016_786_MOESM4_ESM.pdf]

Table S4. The association between selected SNPs and ER negative breast cancer risk in East Asian women.

							Among ER negative cases and all controls					
Chr	Position	SNP	Gene	EA[a]	OA[a]	EAF[a]	Number	beta	OR	se	one-sided p	Homo_p[b]
44 SNPs used for the construction of the polygenic genetic score												
1	10566215	rs616488	PEX14	A	G	0.70	15085	0.1116	1.12	0.0306	1.34E-04	1.97E-01
1	121280613	rs11249433	EMBP1	G	A	0.08	15087	0.0486	1.05	0.0773	2.65E-01	3.61E-01
1	203766331	rs4951011	ZC3H11A	G	A	0.31	15088	0.0790	1.08	0.0307	5.10E-03	3.83E-01
2	19320803	rs12710696	MIR4757	T	C	0.33	11363	0.0284	1.03	0.0354	2.12E-01	7.17E-01
2	121245122	rs4849887	LOC84931	C	T	0.80	15088	0.0855	1.09	0.0356	8.15E-03	5.85E-01
2	202143928	rs10931936	CASP8	T	C	0.29	11364	0.0521	1.05	0.0365	7.70E-02	6.60E-01
2	217905832	rs13387042	TNP1	A	G	0.12	15088	-0.0011	1.00	0.0445	5.10E-01	1.84E-01
2	218296508	rs16857609	DIRC3	T	C	0.60	15084	0.0885	1.09	0.0288	1.06E-03	9.02E-01
3	27416013	rs4973768	SLC4A7	T	C	0.21	15085	0.0745	1.08	0.0346	1.58E-02	1.37E-01
3	30682939	rs12493607	GFBR2	C	G	0.67	11363	0.0848	1.09	0.0359	9.00E-03	5.19E-01
4	175846426	rs6828523	ADAM29	C	A	0.75	11363	0.0156	1.02	0.0375	3.39E-01	1.89E-01
5	1279790	rs10069690	TERT	T	C	0.20	15083	0.1449	1.16	0.0383	7.90E-05	2.22E-02
5	44706498	rs10941679	MRPS30	G	A	0.50	11364	-0.0138	0.99	0.0341	6.57E-01	2.10E-02
5	56031884	rs889312	MAP3K1	C	A	0.55	15085	0.0103	1.01	0.0284	3.59E-01	2.70E-01
5	90732225	rs10474352	LOC100129716	C	T	0.55	15088	0.0936	1.10	0.0298	8.40E-04	5.98E-01
5	158244083	rs1432679	EBF1	C	T	0.65	15088	0.0869	1.09	0.0299	1.87E-03	5.68E-01
6	149608874	rs9485372	TAB2	G	A	0.58	11364	0.1357	1.15	0.0344	3.93E-05	5.13E-01
6	151948366	rs2046210	C6orf97	A	G	0.38	15088	0.3040	1.36	0.0289	0.00E+00	7.67E-03
8	29509616	rs9693444	C8orf75	A	C	0.29	15087	0.0713	1.07	0.0310	1.08E-02	8.23E-01
8	76230301	rs6472903	HNF4G	T	G	0.96	15088	0.0888	1.09	0.0728	1.12E-01	5.71E-01
8	128387852	rs1562430	POU5F1B	T	C	0.83	15088	0.0651	1.07	0.0375	4.11E-02	8.73E-01
9	22062134	rs1011970	CDKN2B-AS1	T	G	0.09	15087	0.1104	1.12	0.0470	9.40E-03	1.21E-01
9	110306115	rs10759243	9q31.2	A	C	0.44	11364	0.0394	1.04	0.0335	1.20E-01	6.82E-01
10	64251977	rs10822013	ZNF365	T	C	0.50	15088	0.0764	1.08	0.0280	3.21E-03	8.71E-01
10	80841148	rs704010	ZMIZ1	T	C	0.32	15085	0.0630	1.07	0.0301	1.81E-02	9.29E-01
10	123093901	rs11199914	10q26.12	C	T	0.62	11364	0.0009	1.00	0.0345	4.90E-01	9.52E-02
10	123337335	rs2981579	FGFR2	A	G	0.46	15088	0.0842	1.09	0.0286	1.65E-03	1.42E-03
11	1941946	rs909116	TNNT3	T	C	0.39	15076	0.0690	1.07	0.0306	1.21E-02	9.63E-01

11	69328764	rs614367	CCND1	T	C	0.01	15086	0.0173	1.02	0.1473	4.54E-01	6.42E-02
11	129473690	rs7107217	BARX2	C	A	0.37	11359	0.1062	1.11	0.0345	1.03E-03	8.63E-01
12	14413931	rs12422552	ATF7IP	C	G	0.29	11362	-0.0112	0.99	0.0378	6.16E-01	3.76E-02
12	28155080	rs10771399	PTHLH	A	G	0.82	15088	0.1315	1.14	0.0366	1.65E-04	3.40E-01
12	96027759	rs17356907	NTN4	A	G	0.76	15088	0.0715	1.07	0.0335	1.64E-02	7.62E-01
12	115836522	rs1292011	MED13L	A	G	0.75	15087	0.1444	1.16	0.0331	6.50E-06	3.64E-01
14	37132769	rs2236007	PAX9	G	A	0.72	15082	0.0667	1.07	0.0317	1.77E-02	6.61E-01
14	91841069	rs941764	CCDC88C	G	A	0.15	15086	0.0538	1.06	0.0391	8.45E-02	7.13E-01
15	91512067	rs2290203	PRC1	G	A	0.51	11364	0.0318	1.03	0.0329	1.67E-01	1.15E-01
16	52586341	rs3803662	LOC643714	A	G	0.63	15086	0.1045	1.11	0.0296	2.04E-04	2.12E-01
16	52599188	rs4784227	LOC643714	T	C	0.27	15088	0.1522	1.16	0.0319	8.80E-07	2.17E-02
16	53855291	rs11075995	FTO	A	T	0.31	11363	0.0420	1.04	0.0360	1.22E-01	4.87E-01
18	24337424	rs527616	LOC728606	G	C	0.71	15087	0.0040	1.00	0.0307	4.48E-01	1.39E-01
19	17394124	rs2363956	ANKLE1	T	G	0.68	15083	0.1159	1.12	0.0310	9.10E-05	3.03E-02
19	18571141	rs4808801	ELL	A	G	0.75	15082	0.0664	1.07	0.0326	2.10E-02	6.19E-01
22	39358037	rs12628403	APOBEC3A	C	A	0.34	15082	0.1085	1.11	0.0359	1.26E-03	9.77E-01
Other SNPs												
1	114448389	rs11552449	DCLRE1B	T	C	0.60	15087	0.0308	1.03	0.0301	1.53E-01	6.84E-01
1	202187176	rs6678914	LGR6	G	A	0.77	11363	0.0432	1.04	0.0398	1.39E-01	3.26E-01
1	204518842	rs4245739	MDM4	C	A	0.05	11362	-0.0156	0.98	0.0777	5.80E-01	4.19E-01
2	172972971	rs2016394	METAP1D	G	A	0.79	11364	-0.0160	0.98	0.0430	6.45E-01	3.89E-01
2	174212894	rs1550623	CDCA7	A	G	0.98	11359	0.1294	1.14	0.1480	1.91E-01	7.15E-01
2	202149589	rs1045485	CASP8	G	C	1.00	15086	-0.3219	0.72	0.4791	7.49E-01	9.94E-01
3	4742276	rs6762644	ITPR1/EGOT	G	A	0.09	15087	0.0313	1.03	0.0506	2.69E-01	7.30E-01
4	106084778	rs9790517	TET2	T	C	0.60	11362	0.0362	1.04	0.0342	1.45E-01	3.65E-01
5	44899885	rs9790879	MRPS30	T	C	0.44	11361	0.0129	1.01	0.0335	3.50E-01	1.28E-01
5	58184061	rs10472076	RAB3C	C	T	0.26	15087	-0.0177	0.98	0.0324	7.07E-01	3.21E-01
5	58337481	rs1353747	PDE4D	T	G	1.00	11364	0.2949	1.34	0.2852	1.51E-01	7.00E-01
6	1318878	rs11242675	FOXQ1	T	C	0.45	15087	-0.0516	0.95	0.0283	9.66E-01	1.85E-01
6	13722523	rs204247	RANBP9	G	A	0.60	15087	-0.0317	0.97	0.0286	8.66E-01	2.31E-02
6	82193109	rs17530068	FAM46A	C	T	0.22	15088	0.0638	1.07	0.0336	2.88E-02	1.77E-01
6	151914113	rs3757318	C6orf97	A	G	0.28	15087	0.1912	1.21	0.0319	9.80E-10	3.99E-01
7	144074929	rs720475	ARHGEF5/NOBO	G	A	0.96	11364	-0.0941	0.91	0.0874	8.59E-01	1.67E-01
8	76417937	rs2943559	HNF4G	G	A	0.09	11364	-0.0685	0.93	0.0610	8.69E-01	5.20E-01

8	128355618	rs13281615	POU5F1B	G	A	0.52	14950	0.0216	1.02	0.0286	2.25E-01	9.36E-01
8	129194641	rs11780156	MIR1208	T	C	0.21	11364	-0.0230	0.98	0.0402	7.17E-01	9.24E-01
9	110888478	rs865686	9q31	T	G	0.93	15088	0.0881	1.09	0.0569	6.10E-02	6.34E-01
10	5886734	rs2380205	ANKRD16	C	T	0.88	11361	-0.0352	0.97	0.0504	7.58E-01	4.49E-01
10	22032942	rs7072776	MLLT10/DNAJ	A	G	0.06	15088	-0.0056	0.99	0.0683	5.33E-01	8.60E-01
10	22315843	rs11814448	DNAJC1	C	A	0.01	15088	0.0208	1.02	0.1446	4.43E-01	6.25E-01
10	64278682	rs10995190	ZNF365	G	A	0.98	15088	-0.0553	0.95	0.0947	7.21E-01	3.33E-01
10	114773927	rs7904519	TCF7L2	G	A	0.07	15087	0.0420	1.04	0.0806	3.01E-01	6.68E-01
10	123352317	rs2981582	FGFR2	A	G	0.33	15086	0.0454	1.05	0.0299	6.45E-02	9.21E-04
11	1909006	rs3817198	LSP1	C	T	0.14	15086	0.1039	1.11	0.0401	4.75E-03	6.72E-01
11	65583066	rs3903072	OVOL1	G	T	0.79	11364	0.0524	1.05	0.0416	1.04E-01	7.64E-01
11	129461171	rs11820646	BARX2	C	T	0.54	15086	0.0413	1.04	0.0288	7.55E-02	9.12E-01
13	32972626	rs11571833	BRCA2	T	A	0.00	15087	0.4610	1.59	0.8818	3.01E-01	7.81E-01
14	68660428	rs2588809	RAD51L1	T	C	0.03	15088	-0.0152	0.98	0.0943	5.64E-01	7.05E-01
14	69034682	rs999737	RAD51B	C	T	1.00	15087	-0.1769	0.84	0.2136	7.96E-01	9.11E-01
14	69039588	rs8009944	RAD51B	C	A	0.73	11363	0.0140	1.01	0.0383	3.57E-01	5.08E-01
16	52548037	rs12443621	TOX3	G	A	0.57	11364	0.0845	1.09	0.0335	5.85E-03	3.85E-02
16	53813367	rs17817449	MIR1972	T	G	0.86	15087	0.0564	1.06	0.0413	8.55E-02	6.91E-01
16	80650805	rs13329835	CDYL2	G	A	0.05	15088	0.1023	1.11	0.0613	4.75E-02	7.24E-01
17	53056471	rs6504950	STXBP4/COX1	G	A	0.91	11364	0.0116	1.01	0.0580	4.21E-01	9.44E-01
18	24570667	rs1436904	CHST9	T	G	0.54	15085	0.0247	1.02	0.0282	1.91E-01	9.14E-01
19	17389704	rs8170	BABAM1	A	G	0.01	15087	0.1071	1.11	0.2338	3.24E-01	4.91E-01
19	44286513	rs3760982	KCNN4/ZNF28	A	G	0.16	15088	0.0099	1.01	0.0392	4.00E-01	9.37E-01
20	32588095	rs2284378	RALY	T	C	0.18	11364	-0.0061	0.99	0.0434	5.56E-01	5.04E-01
21	16520832	rs2823093	NRIP1	G	A	0.96	11364	-0.0145	0.99	0.0805	5.72E-01	1.37E-01
22	29621477	rs132390	EMID1/RHBDD3	C	T	0.00	11364	-0.1391	0.87	0.4057	6.34E-01	5.56E-01
22	40876234	rs6001930	MKL1	C	T	0.25	15087	-0.0402	0.96	0.0323	8.93E-01	2.09E-02

[a]Abbreviations: EA, effect allele; OA, other allele; EAF, effect allele frequency.

[b]Homo_p: p values for homogeneity tests between ER positive and ER negative breast cancer cases.
