# Supplementary material for: Prediction of breast cancer risk based on common genetic variants in women of East Asian ancestry
Source: Breast Cancer Res. 2016 Dec 8;18:124. doi: 10.1186/s13058-016-0786-1 (PMC5146840; doi:10.1186/s13058-016-0786-1)
Supplement: Additional file 5: — is Table S5 presenting ethics committees that approved participating studies. (PDF 59 kb) [file 13058_2016_786_MOESM5_ESM.pdf]

Table S5. Ethics committees that approved participating studies

Study	Country	Approval Committee
Asia Cancer Program (ACP)	Thailand	University Faculty of Medicine Ethics Committee; Khon Kaen University Ethics Committee for Human Research; HRH Princess Maha Chakri Sirindhorn Medical Centre (MSMC) Ethics Committee
Hospital-based Epidemiologic Research Program at Aichi Cancer Center (HERPACC)	Japan	Ethics Committee for Human Genome Study at Aichi Cancer Center
Los Angeles County Asian-American Breast Cancer Case-Control Study (LAABC)	USA	University of Southern California Health Sciences Campus IRB
Malaysian Breast Cancer Genetic Study (MYBRCA)	Malaysia	University Malaya Medical Centre Medical Ethics Committee
Shanghai Breast Cancer Genetic Study (SBCGS)	China	Shanghai Cancer Institute, Shanghai Center for Disease Prevention and Control IRB and Vanderbilt University Medical Center IRB
Seoul Breast Cancer Study (SEBCS)	Korea	Seoul National University College of Medicine/Seoul National University Hospital IRB
Singapore Breast Cancer Cohort (SGBCC)	Singapore	National Health Group (NHG) Domain Specific Review Board (DSRB)
IARC-Thai Breast Cancer Study (TBCS)	Thailand	IARC Institutional Review Board Committee
Taiwanese Breast Cancer Study (TWBCS)	Taiwan	Human Subject Research Ethics Committee/IRB Academia Sinica
Shanghai Breast Cancer Study (SBCS)	China	Shanghai Cancer Institute and Vanderbilt University Medical Center IRB
Shanghai Breast Cancer Survival Study (SBCSS)	China	Shanghai Cancer Institute and Vanderbilt University Medical Center IRB
Shanghai Women’s Health Study (SWHS)	China	Shanghai Cancer Institute and Vanderbilt University Medical Center IRB
